# Effect of physical parameters and temperature on the piezo-electric jetting behaviour of UV-curable photochromic inks

**DOI:** 10.1038/s41598-020-75449-z

**Published:** 2020-11-02

**Authors:** Sina Seipel, Junchun Yu, Vincent A. Nierstrasz

**Affiliations:** Textile Materials Technology, Department of Textile Technology, Faculty of Textiles, Engineering and Business, University of Borås, 501 90 Borås, Sweden

**Keywords:** Materials science, Fluids, Polymers, Materials chemistry, Surface chemistry, Process chemistry

## Abstract

Although resource-efficient processes like inkjet printing have a large potential to foster the development of smart and functional textiles, one bottleneck still is the development of functional inks. To make inkjet printing and UV curing given production techniques for smart and functional specialty products, e.g. photochromic textiles, deepened knowledge about the development, rheological behavior and jetting behavior of functional ink is needed. This paper focuses on the formulation and performance of UV-responsive and UV-curable inkjet inks, which are based on photochromic dyes and their application to produce UV-responsive textiles. Two commercial photochromic dyes—Reversacol Ruby Red (RR) and Sea Green (SG), which represent dyes of the naphthopyran and spirooxazine class, respectively, have been used to develop the inks. The photochromic inks are characterized according to their physical–chemical and rheological properties in respect to temperature. The influence of temperature on the drop formation of the inks in an industrial print head is analyzed using a high-speed camera, which reveals important information regarding challenges in ink jettability. It was found that the dye structure and type used in the ink can influence the jetting behavior of photochromic UV-curable ink. More pronounced temperature sensitivity of dyes can increase the temperature-related effects of drop formation as was observed for SG ink. The printability of the RR and SG inks is framed and underpinned by theoretical calculations of the *Z* number. Discrepancies are observed and discussed between existing theory of ink jettability and visual evaluation of the photochromic ink.

## Introduction

Digital inkjet technology offers a great opportunity for the textile industry not only through the conservation of resources, but also through its high potential to cultivate innovation. Through inkjet printing a flexible textile functionalization process for small batches with a reduced ecological footprint can be realized. Next to the minimized consumption of energy, water and chemicals, the functional chemistry can be applied locally and with complex designs while retaining the textile properties.

Functional finishes on textiles offer an enriching possibility to create smart textile materials and find new fields of application. Until now, a major challenge having to be faced in fostering the technology is to formulate inks beyond the established colorants and instead with advanced and smart functions to cater to future trends and industrial development. Functional inks, which may contain polymers, pigments, dyes, dispersants, additives, binders and other components possess complex rheological behavior^1,2^. This puts high demand on the development and characterization of the ink and challenges a controlled industrial printing process^3^. According to Mendes-Felipe et al*.*^4^ the symbiosis of ink jetting technology and UV-curable materials has large potential as resource-efficient, sustainable and versatile production method for next generation devices and smart solutions.

Drop-on-demand (DOD) inkjet printing is the most common type of inkjet printing, where tiny droplets with a volume of approx. 10 picolitres are precisely deposited on the surface of the printable substrate. As the name implies, in DOD printing a drop is formed when there is a demand for it according to the print pattern. The ink is fired upon an electric signal and a droplet with characteristic tail formation is ejected from the nozzle. Through polarization of the lead zirconium titanate the crystal undergoes distortion creating a pressure pulse in the ink chamber^5^. The Starfire SA Dimatix print head used in this study is a typical industrial print head for textile printing, which features a total number of 1024 nozzles.

Drop formation of an ink is influenced by the properties of the mixture of fluids—viscosity, surface tension, density—and by the velocity and size of the droplet^6^. Electric voltage and pulse shape influence the drop formation related to the nozzle geometrics as well^5,6^. As the ink reservoir is not pressurized, the surface tension in the printer prevents unwanted ink flow from the nozzles when in standby mode. Therefore, pressure initiated by the piezoelectric signal helps to overcome a certain surface tension in order for a drop to be formed at the orifice. The pressure difference, which has to be exceeded, is^7^,

1 $$\Delta P=\frac{2\gamma }{r}$$

where $\gamma$ is the surface tension and $r$ the radius of the nozzle.

The theoretical printability of ink, which has been widely discussed in literature^8–12^ can be calculated by a combination of dimensionless numbers, which depend on various physical–chemical properties of the printable fluid and dimensions of the printing orifice. The Reynolds number $Re$ and the Weber number $We$ specify the relative magnitude of the fluid’s interfacial, viscous and inertial forces^6,9,10^.

2 $$Re=\frac{\upsilon \rho r}{\eta }$$

3 $$We=\frac{{\upsilon }^{2}\rho r}{\gamma }$$

where $\upsilon$ is the velocity, $\rho$ the density and $\eta$ the viscosity. The Reynolds number defines the fluid’s inertia to its viscosity, whereas the Weber number specifies the ratio of inertia to its surface tension.

Fromm ^10^ has developed a solution based on the Navier–Stokes equations^13^ to express the limitations of drop ejection in regards to interfacial, viscous and inertial properties of the fluid^6^.

4 $$Z=\frac{{(\gamma \rho r)}^{1/2}}{\eta }= \frac{1}{Oh}=\frac{Re}{{We}^{1/2}}$$

$Z$ is the inverse of the Ohnesorge number $Oh$ and is defined as the ratio of the Reynolds number and the square root of the Weber number; also known as Laplace number $La$.

**Figure 1:**
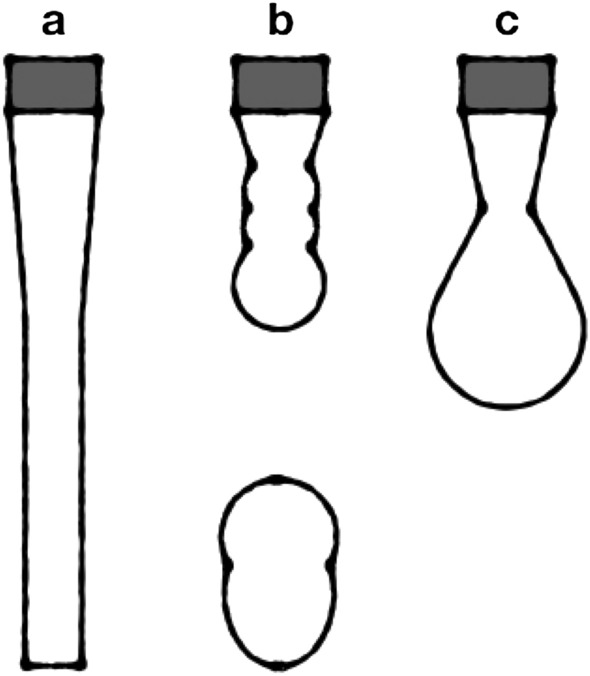
Scheme of (**a**) continuous fluid flow, (**b**) satellite drops and (**c**) stable drop with tail.

The initial specification by Fromm^10^ that $Z>2$ is required for the ejection of stable droplets was revised and updated by Derby^14^ to an acceptable range of 1 < *Z* < 10. The formation of stable droplets implies single droplets with tail formation (c) as seen in Fig. 1. If these conditions cannot be met, so-called satellite droplets (b) will be formed, which impede print quality.

Looking closer into the effects on how a droplet with tail is formed, a cylindrical fluid shape flowing out of the nozzle is assumed in the beginning of the process. During the process a droplet develops from the cylindrically shaped fluid flow and forms a filament combining fluid cylinder and droplet. With continuous approach of the droplet towards the solid surface the filament eventually breaks and a flying droplet is formed^15^. At flight a spherical droplet with a specific and constant volume will form before landing on the printable substrate.

This paper focuses on the exploration of the jettability of UV-curable photochromic inks and their drop formation as function of temperature and its effect of changed physical fluid properties on the drop formation. The results entail empirical data, which is embedded in and confirmed using theoretical calculations based on the dimensionless numbers *We*, *Re* and *Z*.

## Materials and methods

### Ink formulation

In the formulation of photochromic UV-curable inkjet inks two commercial heterocyclic spiro-compounds are used. Reversacol Ruby Red (RR), a naphthopryan-type and Reversacol Sea Green (SG), a spirooxazine-type dye from Vivimed Labs, UK are combined with a UV-curable varnish to fit print head specifications. Pure compounds available at Sigma-Aldrich have a molecular weight of approx. 330 g/mol for spirooxazines and 220 g/mol for naphthopyrans. The dye concentration in the designed inkjet inks is 4 g/l. The UV-curable varnish consists of dipropylene glycole diacrylate monomers (DPGDA), amine modified polyetheracrylate oligomers (Ebecryl 81) supplied by Allnex SA/NV, Belgium and a UV-LED photo-initiator (Genocure TPO-L) supplied by Rahn AG, Switzerland. The ratio of component parts for monomer/oligomer/photo-initiator is 21/1/1. For dissolution and homogeneous dye dispersion solvents are used in the ink formulation, which are removed after stirring by vacuum pumping. Ethyl acetate, 99.9% (Chromasolv Plus) is used for RR and hexane, ≥ 97% (Chromasolv HPLC) for SG, both purchased from Sigma-Aldrich. A mixture of isomers, di-propylene glycol methyl ether acetate (DPGMEA) purchased from Sigma-Aldrich, is used as standard cleaning fluid for the Starfire SA print head and serves as a reference fluid for the evaluation of the ink jettability of the photochromic inks. DPGMEA has a surface tension of 31.1 mN/m and a viscosity of 4.8 mPa s at 20 °C.

### Rheological and physical properties

The viscosity was measured using a rheometer Physica MCR500 from Paar Physica with a double gap cylindrical cell. The viscosity was acquired at the maximum shear rate of the rheometer of 10,000 1/s at a temperature sweep from 20 to 40 °C and 40–20 °C, which was repeated twice. A shear rate of 10,000 1/s simulates the conditions upon jetting as the estimated shear rate at the nozzle tip of a Dimatix print head could reach 40,000 1/s. The surface tension of the ink fluids was measured using an optical tensiometer Attension Theta from Biolin Scientific. Three individual measurements were made with the pendant drop method and drop size of 6 μl at 22 °C.

### Visual evaluation of ink jettability

The two photochromic inks and DPGMEA are jetted with a Starfire SA print head from Fujifilm Dimatix, which prints with a resolution of 400 dpi and in three different grey scales. The radius of the orifices featured in the print head is estimated to 10 μm and the printable range is specified with η = 8–20 mPa s (recommended 10–14 mPa s) and γ = 20–35 mN/m. Visual analysis of the drop formation is made with a Uridium drop watcher high-speed camera. Jetting results at a head voltage of 110 V, max. frequency of 14 kHz and with a waveform of three pulses with increasing amplitudes of 50/70/90 V resulting in three dots per drop (DPD). The photochromic inks’ behaviour as function of temperature was analysed in detail in regards to drop volume and drop velocity at a delay of 200 μs, which is optimal for picture processing. The temperature was increased in steps of 5 °C between 25 and 40 °C with a waiting time of 30 min for conditioning.

For comparison of the visual analysis with the fluids’ theoretical jetting behavior, the printability of the fluids RR ink, SG ink and DPGMEA are calculated based on their physical properties using Eqs. (2)–(4).

## Results and discussion

### Ink characterization

**Figure 2:**
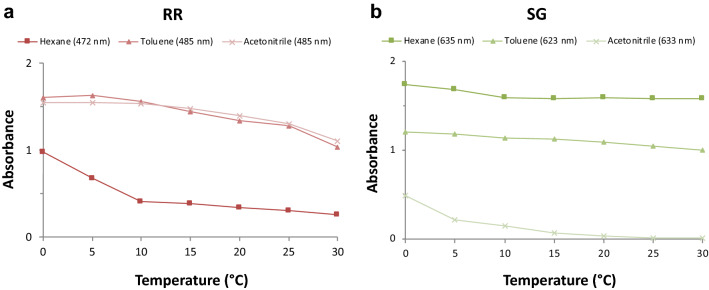
Temperature and solvent dependency of absorbance of (**a**) Ruby Red (RR) and (**b**) Sea Green (SG) photochromic dyes.

UV-curable photochromic inks are specialty inks, which are designed for photochromic sensory applications on textile surfaces. The UV-curable photochromic inks RR and SG ink are characterized according to their absorbance for the expected photochromic color effects and to their physical properties in order to match print head requirements. Color effects of photochromic dyes are inherent to the respective dye class, influenced by solvent polarity and temperature with generally higher thermal conversion at higher temperatures^16,17^. Naphthopryan dyes are inherently more stable and less temperature-sensitive than spirooxazine dyes^18–20^. As can be seen in Fig. 2, RR has stronger absorbance in polar solvents as acetonitrile, whereas SG has stronger absorbance in non-polar as hexane. Both these specific commercial photochromic dyes exhibit rather low temperature-dependence, which is desirable for the application at varying temperatures for wearable or other non-wearable smart applications.

**Figure 3:**
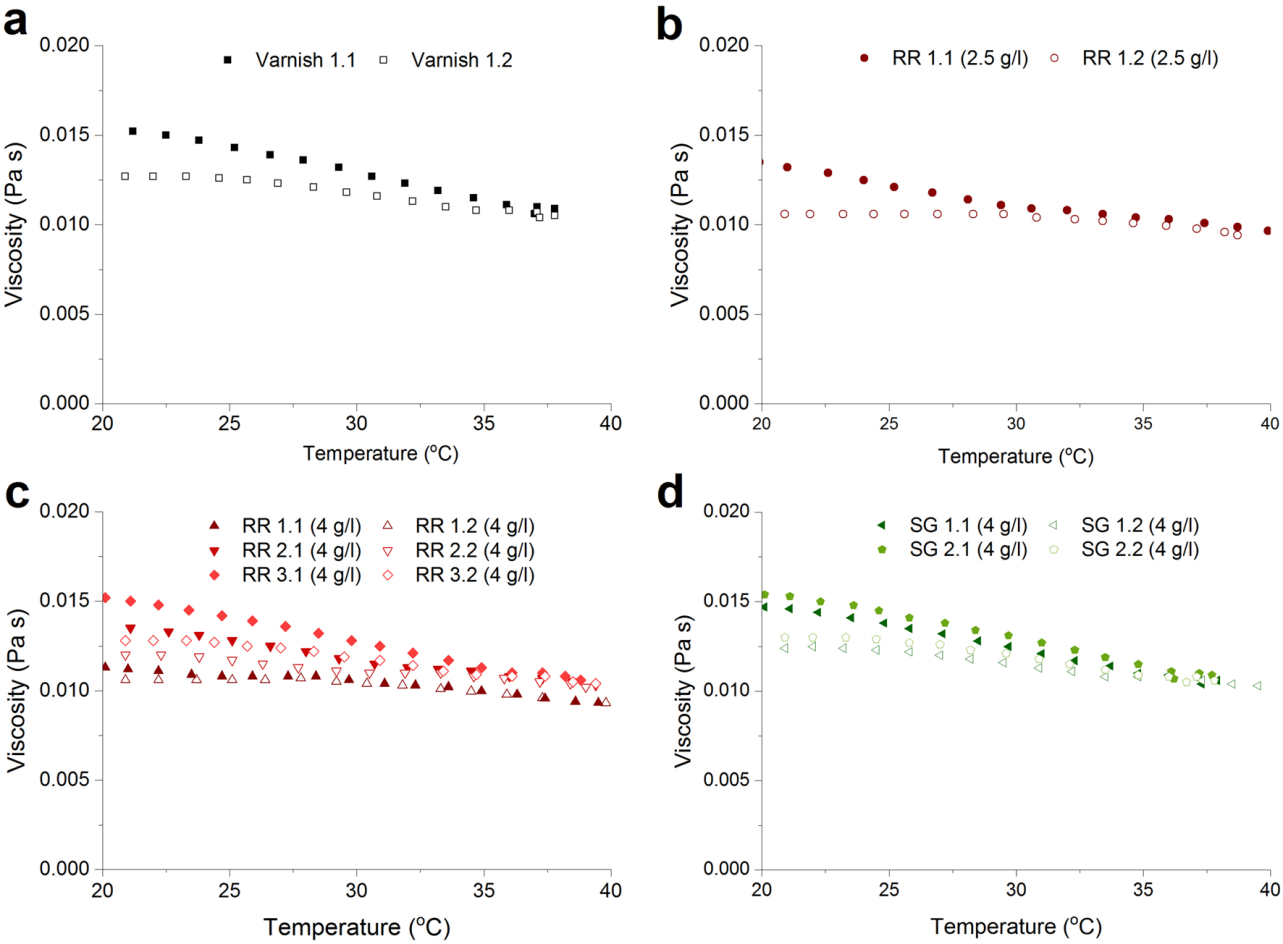
Influence of temperature on the viscosity of UV-curable varnish (**a**) and photochromic inks with 2.5 g/l of RR (**b**), 4 g/l of RR (**c**) and 4 g/l SG (**d**) measured in two sweeps each (0.1/0.2).

In Fig. 3, the typical behavior of the inversely proportional relation between temperature and a substance’s viscosity is seen. The increase in temperature has a thinning effect on the different ink formulations. The viscosity of both the varnish (Fig. 3a) and the photochromic inks (Fig. 3b–d), irrespective of dye concentration and dye type, decreases with increasing temperature. The viscosity has been measured twice at a temperature sweep from 20 to 40 °C for the formulations after a cooling phase. It can be seen that for the second set of measurements the viscosity is lower at the starting temperature for all ink formulations, which is a characteristic of the inks’ inertia. On average a 20 °C increase in temperature lowers the viscosity from 0.014 to 0.010 Pas, which approximates a decrease of 0.001 Pas per 5 °C. The change in viscosity as function of temperature seems to be characteristic for the UV-curable varnish, which is neither influenced by the type or concentration of photochromic dye.

In terms of physical properties as viscosity and surface tension, RR and SG ink are similar with a viscosity of ca. 14.5 mPas and a surface tension of 31 mN/m as listed in Table 1. SG ink has a lower density with 1024 kg/m^3^ compared to RR ink with 1077 kg/m^3^. Despite differences in density, the resulting *Z*, which determines the ink printability, reaches similar values with 1.28 for RR ink and 1.24 for SG ink. In respect to drop velocity and calculated dimensionless numbers *Re* and *We,* RR and SG inks distinguish themselves more from one another. Whereas RR ink drops reach a velocity of 4.35 m/s at a delay of 200 μs, SG ink drops are slower with a velocity of 2.39 m/s. This also has an impact on the Reynolds and Weber number, which both are higher for RR ink than SG ink.

**Table 1:** Physical properties and dimensionless numbers of ink fluids at 22 °C.

Ink fluid	Density (kg/m^3^)	Viscosity (Pa s)	Surface tension (N/m)	Velocity at delay of 200 μs (m/s)	Reynolds number (Re)	Weber number (We)	Inverse (Z) of Ohnesorge number (Oh)
RR	1077 ± 31.8	0.0143 ± 0.001	0.031 ± 0.0002	4.35 ± 0.14	3.28 ± 0.11	6.58 ± 0.44	1.28
SG	1024 ± 20.2	0.0144 ± 0.0004	0.0309 ± 0.0002	2.39 ± 0.4	1.71 ± 0.28	1.92 ± 0.63	1.24
DPGMEA	980	0.0048 ± 0.0001	0.0311 ± 0.0017	6.73 ± 0.1	13.74 ± 0.2	14.27 ± 0.42	3.64

To compare the jetting behavior of the ink formulations with a standard fluid, DPGMEA is used. Compared with the photochromic inks, DPGMEA has a similar density of 980 kg/m^3^, a similar surface tension of 31.1 mN/m, but a three-fold lower viscosity with 4.8 mPas. The lower viscosity affects the dimensionless numbers *Re*, *We* and *Z* with increased values. *Z* of DPGMEA reaches a nearly three-fold higher value with 3.64 compared to the photochromic ink formulations. According to the original specification of ink jettability of Fromm^10^ that *Z* > 2, this would mean that DPGMEA is printable, but RR and SG inks are not. According to Reis and Derby’s^21^ further refinement, however, both photochromic inks and DPGMEA are classed as printable with stable drop formation.

### Visual jetting performance

**Figure 4:**
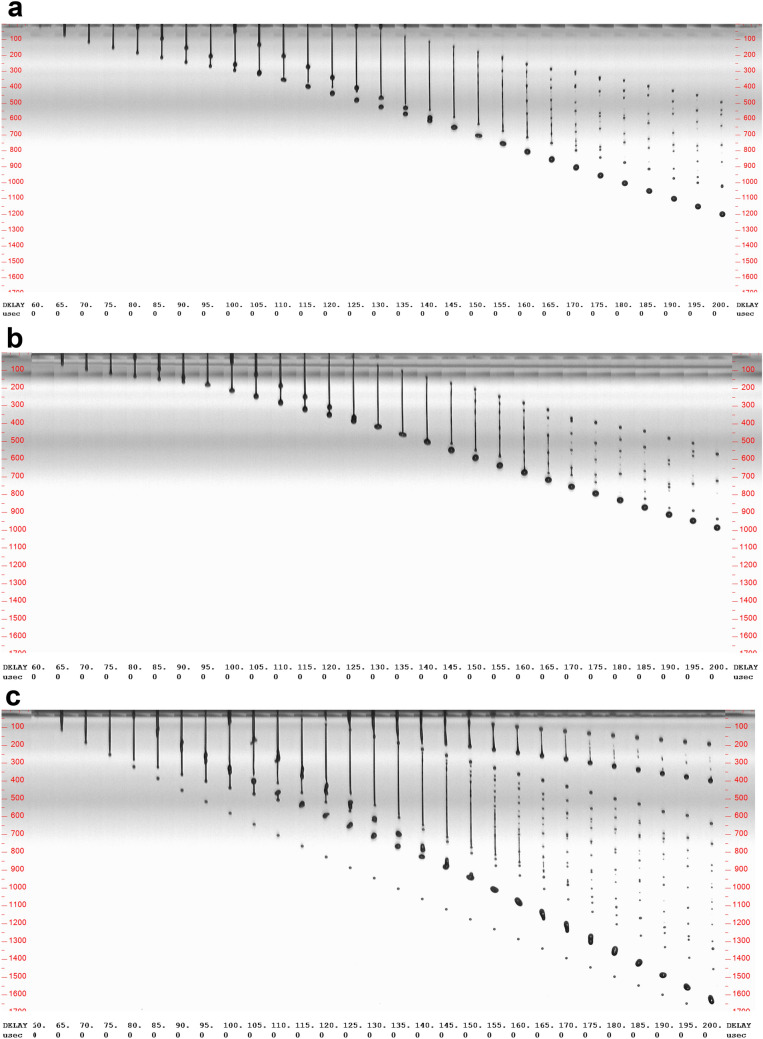
Representative photo sequences of drop formation of (**a**) RR ink, (**b**) SG ink and (**c**) DPGMEA at a delay between 60 and 200 μs at 35 °C.

The jetting behavior as function of temperature of the photochromic inks in relation to DPGMEA as a standard fluid is visually analyzed at a delay sweep between 50 and 200 μs. When ink is printed on a substrate, the substrate will be positioned at a distance of 2–3 mm from the nozzle plate, which means that according to the jetting sequences (Fig. 4a,b), UV-curable ink drops have travelled half way towards the substrate at a delay of 200 μs at 35 °C. Visual analysis is complemented by calculations using the dimensionless numbers *Re*, *We* and *Z*, which determine the printability of ink based on the physical properties of the fluid viscosity η, surface tension γ and drop velocity ν. The varnish, RR and SG inks exhibit a shear-thinning behavior as function of shear rate from 0.1 to 10,000 1/s at 20 °C (Figure S1 in Supplementary Material).

As seen in the representative photo sequences, after firing of two ink drops of RR ink and SG ink (Figures S2 and S3 in Supplementary Material, respectively), they initially are attached to the ligament, i.e. tail, until the tail dissipates in many small drops, i.e. satellite drops. For DPGMEA, unstable drop formation without definition of ligament and drop occurs from the start (Figure S4 in Supplementary Material).

### Effect of temperature

Changes in velocity as a result of change in temperature have an impact on the drop formation, which is seen in Figures S2–S4 (Supplementary information), but also in calculated *Z* numbers (Table 2). Changes in *Z*, eventually mean that also the expected print quality is affected, which however is not experimentally evaluated in this study. The impact of temperature on the surface tension of the fluids is neglected as of very small changes in the range of room temperature to 40 °C. Hence, γ is constant in the calculations of the Weber number *We*.

**Table 2:** Temperature dependence of physical properties and dimensionless numbers of photochromic inks.

Ink fluid	Temperature (°C)	Viscosity (Pa s)	Volume (pL)	Velocity at delay of 200 μs (m/s)	Reynolds number (Re)	Weber number (We)	Inverse (Z) of Ohnesorge number (Oh)
RR ink	25	0.0121 ± 0.0014	35.3 ± 7	4.54 ± 0.02	4.04 ± 0.01	7.17 ± 0.05	1.51
	30	0.0114 ± 0.0009	32.3 ± 1.2	5.27 ± 0.12	5 ± 0.11	9.66 ± 0.42	1.61
	35	0.0092 ± 0.0040	32.0 ± 1.4	6.04 ± 0.06	7.09 ± 0.07	12.63 ± 0.24	1.99
	40	0.0100 ± 0.0006	37.0 ± 1.7	6.60 ± 0.06	7.09 ± 0.06	15.12 ± 0.27	1.82
SG ink	25	0.0133 ± 0.0006	31 ± 5	2.43 ± 0.13	1.87 ± 0.1	1.96 ± 0.2	1.34
	30	0.0123 ± 0.0010	29.7 ± 2.9	4.17 ± 0.12	3.48 ± 0.1	5.76 ± 0.33	1.45
	35	0.0111 ± 0.0003	31.7 ± 1.2	4.95 ± 0.1	4.58 ± 0.09	8.11 ± 0.32	1.61
	40	0.0105 ± 0.0002	27.3 ± 1.2	5.54 ± 0.13	5.42 ± 0.12	10.19 ± 0.47	1.7

When applying Eqs. (2)–(4) with available experimental data, i.e. temperature-dependent viscosity and velocity values, it can be understood that the jettability improves based on increasing *Z* values. For RR ink *Z* increases from 1.27 at room temperature to 1.98 at 35 °C. For higher temperatures than 35 °C the *Z* decreases again to 1.82, which suggests that 35 °C is an optimal printing temperature for the ink formulation.

Despite similar physical properties, i.e. surface tension and viscosity as presented in Table 1, of RR and SG inks, visual analysis reveals distinct jetting behavior of the photochromic inks. At room temperature (22–23 °C) and 25 °C droplets of SG ink have a remarkably lower velocity (ca. 2 m/s) with 2.4 m/s than RR ink droplets with 4.5 m/s. At higher temperatures the difference is smaller (ca. 1 m/s) with ν_SG_ = 5–5.5 m/s and ν_RR_ = 6–6.6 m/s. An explanation for the difference in jettability at lower temperatures can be due to the more bulky and rigid structure and higher molecular weight of spirooxazines^22,23^, which impedes jetting of the ink from the orifice. At temperatures from 35 °C the lower viscosity of the varnish enables more flexibility for the dye to move and hence smoother jetting flow. This effect is more prominent for SG ink as spirooxazines are more temperature-sensitive than naphthopyrans^18–20^. Zhang et al.^24^ studied the effects of reactive dye structures on the surface tensions and viscosities of dye solutions (waterborne). They found out that relatively large dye molecules can significantly increase the viscosity of the dye solution, i.e. the dye structure as well as molecular weight has an impact on the viscosity of the ink solution. The more bulky and rigid structure as well as higher molecular weight of spirooxazines may have increased van der Waals forces between the molecules therefore affect the viscosity and jetting behaviour of SG ink.

Another critical difference between the ink formulations RR and SG is the drop volume. Whereas, temperature does not have a significant and coherent effect on the drop volume, the type of dye has. SG ink exhibits generally smaller drops with 27–32 pL compared with RR ink, which jets larger drops of 32–37 pL. The reason for a smaller drop volume could also be owing to the difference in molecular structure of the dyes. As mentioned earlier, spirooxazines are more rigid than naphthopyrans and non-planar in their inactivated state, which is the case upon jetting. Spirooxazines also have a higher molecular weight than naphthopyrans, which however is not specified for the commercial dyes RR and SG. As the difference in molecular structure between the two dyes has shown to have a distinct effect on the color kinetics of photochromic prints using RR and SG^25^, it is likely to affect drop formation and can cause a bottleneck at the orifice resulting in smaller drop volumes.

### Non-Newtonian effects

Most inkjet inks exhibit non-Newtonian and viscoelastic behavior as they either contain particles, large molecules, surfactants or other additives^1,26^ or as in the case of DPGMEA, RR and SG inks, consist of a mixture of isomers, monomers and oligomers. In Fig. 4a,b, it can be seen that despite the ink formulations RR and SG ink being classified as jettable fluids within the range 1 < *Z* < 10 according to Derby^27^, satellite drops are formed. But according to the classification, a value of *Z* > 10 would result in the formation of satellite drops. The observed formation of satellite drops of the ink formulations RR and SG ink presumably is due to the more complex rheological effects of non-Newtonian fluids and their impact on drop formation^15,28,29^. However, theoretical models are based on Newtonian fluids. For non-Newtonian fluids, specific adjustments in the waveform were successful in reducing satellite drops and improving print quality^30^, which might be a promising investigation for UV-curable acrylate inks. Jo et al*.*^31^ found that increasing the viscosity of a fluid, targeting a decrease in *Z*, stabilizes the tail formation and reduces the risk for satellite drops for Newtonian fluids. Such an increase in viscosity must, of course, be within the possible range for the specific print head. For example, a photochromic RR ink with decreased monomer amount (with 7/1/1-parts of monomer/oligomer/photo-initiator) will result in a viscosity of 22 mPas and a surface tension of 31.9 mN/m. Whereas the surface tension of the ink is within the required range, the viscosity exceeds the specifications of the Starfire SA print head as stated earlier. Calculation of the *Z* value with an assumed similar drop velocity as for the RR ink with 4.35 m/s and similar density of 1080 kg/m^3^ results in a *Z* = 0.8, which predicts that the fluid is not printable. A *Z* < 1 would prevent drop ejection due to viscous dissipation^32^. Despite the fact that this formulation is non-jettable, it shows that an increase in viscosity can decrease the *Z* value.

According to print head specifications of the Starfire SA, the physical properties of DPGMEA are partially challenging. Whereas a surface tension of 31.1 mN/m is in the jetting range, the three-fold lower viscosity than the photochromic inkjet inks of 4–5 mPas is below the recommended range for the Starfire SA print head. This would make DPGMEA not an ideal fluid for good print quality. However, according to calculations based on the physical properties of DPGMEA with *Re* = 13.74, *We* = 14.27 and a resulting *Z* = 3.64, the fluid is within the printable region of 1 < *Z* < 10. It is though obvious from the photo sequence (Fig. 4c and Fig. S4 in Supplementary Material) that due to the low viscosity of the fluid uncontrolled dripping results and satellite drops dominate. It can also be seen that temperature does not have a decisive effect in changing the jetting behavior as compared to photochromic inks, where it is obvious that with increasing temperature the tail becomes longer, a distinct drop forms earlier and the velocity increases (Figures S2 and S3). Another reason for the discrepancy between theoretical calculations and the visual assessment of a real ink in an industrial printhead, is the detection of viscoelastic behavior of inks, which occurs in a very short time frame. Using mechanical oscillation rheometers, these effects are not detectable in viscosity measurements^1^ and therefore, visual assessment in industrial printheads reveals important challenges for the formulation of functional inks.

### Tail formation

The length of the formed tail upon jetting as mentioned above varies as effect of temperature, but also between RR and SG inks as seen in Fig. 4, Figs. S2 and S3 in Supplementary Material. SG ink tends to exhibit shorter tails than RR ink, with generally increasing length with higher temperature. This visual observation is contradictory to the improved calculated *Z* value as a result of temperature, as a longer tail is supportive of the formation of satellite drops. Vice versa, a short tail and an optimum viscoelastic behavior of the fluid will lead to that the tail is instantly pulled into the drop after ejection and jet breakup at the nozzle^33^. If the formed tail separates from the drop head this is either caused by Rayleigh-Plateau instability or end pinching^34^. The tail length is predominantly influenced by the printable fluid’s density, where a small increase in density may reduce the tail length significantly^35^. But also, a decrease in viscosity can reduce the tail length non-linearly of polymer-loaded ink solutions. The decrease in viscosity of polymer-containing inks in the production of scaffolds to stabilize tail formation^35^ suggests the opposite trend compared to an increase in viscosity of Newtonian fluids proposed by Jo et al.^31^. And polystyrene-based viscoelastic fluids have shown distinct jetting behavior with differences in ligament formation despite nearly equal low shear rate viscosities^1^. As an increase in viscosity of the photochromic ink negatively influenced the calculated *Z* value, change in density in the ink formulation may help to stabilize drop formation by achieving shorter tail length. However, although SG ink has most stable drop formation with shortest tail at 25 °C, drop velocity at this temperature with 2.4 m/s is very low, which impeded printing on a textile substrate. Therefore, not just in theoretical calculations but also in practice, it could be shown that temperature is an important means of improving ink jettability and resulting print quality. For inkjet printing of photochromic inks on polyester fabric the print head temperature was set to 35 °C^25,36^. However, these studies did not include the analysis of resolution and sharpness of the printed patterns, which possibly improves with more stable drop formation.

### Jet breakup

The formation of satellite drops, despite measured and calculated values for stable drop formation, necessitates the discussion in relation to breakup of liquid jets. Several factors influence the behavior of fluids upon breakup of liquid jets, which is decisive for how a drop is formed and the resulting print quality. Although the print quality is not subject to this study, it is known that the instability and breakup of a liquid jet into fine secondary drops while travelling to the printable substrate, results in loss of print quality^37,38^. Vastly studied phenomena in primary and secondary breakup of jets are distinguished with four modes of primary breakup, initially observed by Ohnesorge^39^ and Reitz^40^. Primary breakup occurs closer to the nozzle plate, where larger ligaments and drops detach from a liquid jet. With increasing perturbation effects in primary breakup, the Rayleigh, first and second wind induced and atomization mode are distinguished^41,42^. According to the definitions, RR and SG inks mainly exhibit Rayleigh behavior, where a liquid jet is disrupted by capillary instability caused by axisymmetric perturbations. Drops, which are formed have similar or larger diameter as the jet itself. DPGMEA shows larger instability with agreement of what is defined as first wind induced breakup, where perturbations on the jet surface are present, formed drops are smaller, vary more in size distribution and satellite drops can occur in between main drops.

In secondary breakup further deformation and breakup of the larger ligaments and drops into smaller ones continues until a stable drop is formed^41^. Here, for fluids with a Weber number below the critical value of *We *≈ 12, drops are impacted by oscillation, which cause deformation and eventually vibrational breakup^43,44^. This is the case for SG and RR ink, although drops of SG ink are more stable with a lower *We* of 1.9 than for RR ink with *We* = 6.6. DPGMEA has a *We* of 14.3, which is classified as bag breakup (*We* > 12), where drops initially deform into a spherical cap and then to a flat disk^43–46^.

## Conclusion

This paper points out the discrepancy between theory of jetting of ink, which is based on Newtonian fluids and its application for functional inkjet inks with complex non-Newtonian behavior. The rheology data does not reflect the jetting situation at high shear rate, which exists during the actual printing process. Although calculation of the dimensionless numbers *We*, *Re* and *Z* categorizes ink printability with stable drop formation of RR and SG inks, visual analysis displays flaws and the formation of satellite drops. Phenomena affecting the jettability of UV-curable photochromic ink as non-Newtonian and viscoelastic fluid behavior, tail formation and jet breakup to explain the formation of satellite drops of different ink formulations are discussed. Temperature is a main factor in changing the jetting behavior of naphthopryan- and spirooxazine-based UV-curable photochromic inks. Higher temperature increases drop velocity and improves the theoretical printability of the ink expressed in the *Z* number. Eventually, a temperature of 35 °C is preferable for ink jetting of both RR and SG ink on a substrate. The structure and type of the dye can influence the jetting behavior of photochromic UV-curable ink, which supports similar observations on waterborne ink. More pronounced temperature sensitivity of dyes can increase the temperature-related effects of drop formation.

By analyzing the printing process using an industrial printhead with a high-speed camera, it is possible to unravel difficulties in drop formation despite theoretical and practical printability. Therewith, the design of functional inks, exceeding photochromic inks, can be optimized and eventually facilitate the production of smart and functional textiles with inkjet printing and UV curing as given production techniques.

## Supplementary information

Supplementary Figures.

## References

[1] Hoath S (2009). Links between ink rheology, drop-on-demand jet formation, and printability. J. Imaging Sci. Technol..

[2] Magdassi, S. Ink requirements and formulations guidelines. In *The Chemistry Of Inkjet Inks* (ed. Magdassi, S.) 19–41 (World Scientific Publishing, 2010).

[3] Jeong KM, Kim SG, Won JM, Lee YK, Koseki KI (2017). Rheological properties of UV-curable ink—Influence of the pre-polymer and colorant. J. Photopolym. Sci. Technol..

[4] Mendes-Felipe C, Oliveira J, Etxebarria I, Vilas-Vilela JL, Lanceros-Mendez S (2019). State-of-the-art and future challenges of UV curable polymer-based smart materials for printing technologies. Adv. Mater. Technolog..

[5] Liou T-M, Chan C-Y, Shih K-C (2010). Effects of actuating waveform, ink property, and nozzle size on piezoelectrically driven inkjet droplets. Microfluid. Nanofluid..

[6] Liu Y-F, Tsai M-H, Pai Y-F, Hwang W-S (2013). Control of droplet formation by operating waveform for inks with various viscosities in piezoelectric inkjet printing. Appl. Phys. A.

[7] White, F. M. *Fluid Mechanics* (McGraw-Hill, 1994).

[8] Saunders RE, Derby B (2014). Inkjet printing biomaterials for tissue engineering: Bioprinting. Int. Mater. Rev..

[9] Derby B (2010). Inkjet printing of functional and structural materials: Fluid property requirements, feature stability, and resolution. Annu. Rev. Mater. Res..

[10] Fromm JE (1984). Numerical calculation of the fluid dynamics of drop-on-demand jets. IBM J. Res. Dev..

[11] Jang D, Kim D, Moon J (2009). Influence of fluid physical properties on ink-jet printability. Langmuir.

[12] Derby B (2011). Inkjet printing ceramics: From drops to solid. J. Eur. Ceram. Soc..

[13] Łukaszewicz, G. & Kalita, P. *Navier–Stokes Equations: An Introduction with Applications* (Springer, 2016).

[14] Derby B, Reis N (2003). Inkjet printing of highly loaded particulate suspensions. MRS Bull..

[15] Huang, H. *Non-Newtonian Effects on Ink-Jet Droplet Formation* (York University, 2005).

[16] Billah SMR, Christie RM, Morgan KM (2008). Direct coloration of textiles with photochromic dyes. Part 2: The effect of solvents on the colour change of photochromic textiles. Color. Technol..

[17] Salemi-Delvaux C, Luccioni-Houze B, Baillet G, Giusti G, Guglielmetti R (1995). Effect of photodegradation on the thermal bleaching rate constant of photochromic compounds in spiro[indoline-pyran] and spiro[indoline-oxazine] series. J. Photochem. Photobiol. A: Chem..

[18] Viková, M. *Photochromic Textiles* PhD thesis, Heriot-Watt University, (2011).

[19] Little AF, Christie RM (2011). Textile applications of photochromic dyes. Part 3: Factors affecting the technical performance of textiles screen-printed with commercial photochromic dyes. Color. Technol..

[20] Aldib M, Christie RM (2013). Textile applications of photochromic dyes. Part 5: Application of commercial photochromic dyes to polyester fabric by a solvent-based dyeing method. Color. Technol..

[21] Reis N, Derby B (2011). Ink jet deposition of ceramic suspensions: Modeling and experiments of droplet formation. MRS Proc..

[22] Billah SMR, Christie RM, Shamey R (2008). Direct coloration of textiles with photochromic dyes. Part 1: Application of spiroindolinonaphthoxazines as disperse dyes to polyester, nylon and acrylic fabrics†. Color. Technol..

[23] Periyasamy AP, Vikova M, Vik M (2017). A review of photochromism in textiles and its measurement. Text. Prog..

[24] Zhang K (2019). Effects of reactive dye structures on surface tensions and viscosities of dye solutions. J. Mol. Liq..

[25] Seipel S (2019). Color performance, durability and handle of inkjet-printed and UV-cured photochromic textiles for multi-colored applications. Fiber. Polym..

[26] Son Y, Kim C (2009). Spreading of inkjet droplet of non-Newtonian fluid on solid surface with controlled contact angle at low Weber and Reynolds numbers. J. Nonnewton. Fluid Mech..

[27] Derby B (2015). Additive manufacture of ceramics components by inkjet printing. Engineering.

[28] Ren, Y., Koh, K. & Zhang, Y. Synthesis of functional materials by non-newtonian microfluidic multiphase system. In *Advances in Microfluidics—New Applications in Biology, Energy, and Materials Sciences* (ed. Yu, X.-Y.) (Intech Open, 2016).

[29] Ren Y, Liu Z, Shum HC (2015). Breakup dynamics and dripping-to-jetting transition in a Newtonian/shear-thinning multiphase microsystem. Lab Chip.

[30] Morrison, N. F. & Harlen, O. Inkjet printing of non-Newtonian fluids. In *International Conference on Digital Printing Technologies* (2011).

[31] Jo BW, Lee A, Ahn KH, Lee SJ (2009). Evaluation of jet performance in drop-on-demand (DOD) inkjet printing. Korean J. Chem. Eng..

[32] Reis N, Derby B (2000). Ink jet deposition of ceramic suspensions: Modeling and experiments of droplet formation. MRS Proc..

[33] Zapka W (2017). Handbook of Industrial Inkjet Printing: A Full System Approach.

[34] Driessen, T. & Jeurissen, R. Drop formation in inkjet printing. In *Fundamentals of Inkjet Printing: The Science of Inkjet and Droplets* (ed. Hoath, S. D.) 93–116 (Wiley VCH, 2015).

[35] Basu B, Basu B (2017). Fundamentals of scaffolds fabrication using low temperature additive manufacturing. Biomaterials for Musculoskeletal Regeneration: Concepts.

[36] Seipel S (2018). Inkjet printing and UV-LED curing of photochromic dyes for functional and smart textile applications. RSC Adv..

[37] Rozhkov AN (2005). Dynamics and breakup of viscoelastic liquids (a review). Fluid Dyn..

[38] Bazilevskii AV, Meyer JD, Rozhkov AN (2005). Dynamics and breakup of pulse microjets of polymeric liquids. Fluid Dyn..

[39] Ohnesorge WV (1936). Die Bildung von Tropfen an Düsen und die Auflösung flüssiger Strahlen. ZAMM J. Appl. Math. Mech./Zeitschrift für Angewandte Mathematik und Mechanik.

[40] Reitz, R. D. *Atomization and other breakup regimes of a liquid jet* PhD thesis, Princeton University (1978).

[41] Kékesi, T. *Scenarios of drop deformation and breakup in sprays*, Kungliga Tekniska högskolan (2017).

[42] Liu H (1999). Science and Engineering of Droplets: Fundamentals and Applications.

[43] Pilch M, Erdman CA (1987). Use of breakup time data and velocity history data to predict the maximum size of stable fragments for acceleration-induced breakup of a liquid drop. Int. J. Multiphase Flow.

[44] Faeth GM, Hsiang LP, Wu PK (1995). Structure and breakup properties of sprays. Int. J. Multiphase Flow.

[45] Dai Z, Faeth GM (2001). Temporal properties of secondary drop breakup in the multimode breakup regime. Int. J. Multiphase Flow.

[46] Krzeczkowski SA (1980). Measurement of liquid droplet disintegration mechanisms. Int. J. Multiphase Flow.

