# Supplementary material for: Effect of physical parameters and temperature on the piezo-electric jetting behaviour of UV-curable photochromic inks
Source: Sci Rep. 2020 Nov 2;10:18841. doi: 10.1038/s41598-020-75449-z (PMC7606605; doi:10.1038/s41598-020-75449-z)
Supplement: Supplementary file 1 — Supplementary Figures. [file 41598_2020_75449_MOESM1_ESM.docx]

**Supplementary material**

**Effect of physical parameters and temperature on the piezo-electric jetting behaviour of UV-curable photochromic inks**

Sina Seipel*, Junchun Yu and Vincent A. Nierstrasz

Textile Materials Technology, Department of Textile Technology; Faculty of Textiles, Engineering and Business; University of Borås; 501 90 Borås, Sweden.

*Author to whom correspondence should be addressed:

E-Mail: sina.seipel@hb.se;

Tel.: +46-33-435 4191.

**S1. Rheology of photochromic UV-curable ink**

For inkjet printing inks with a shear-thinning behavior are desired. Figure S1 shows the shear-thinning viscosity of individual batches of the varnish and photochromic inks RR and SG ink as function of shear rate between 0.1 and 10000 1/s at 20 °C.


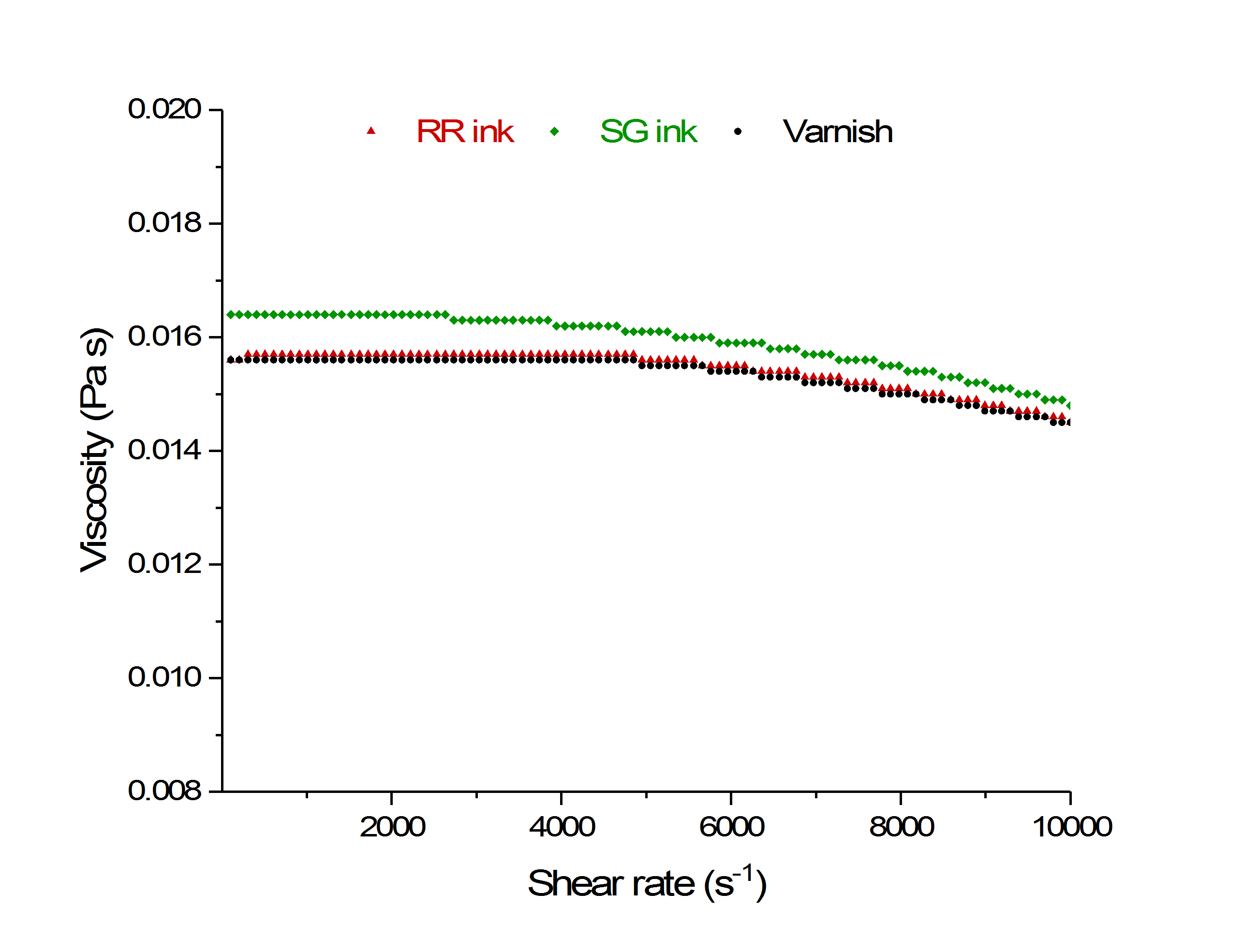


Figure S1: Viscosity of RR ink, SG ink and varnish as function of shear rate at 20 °C.

**S2. Visual assessment of drop formation at different temperatures**

Drop formation is affected by temperature, which is seen in representative photo sequences upon jetting of RR ink (Figure S2), SG ink (Figure S3) and DPGMEA (Figure S4) at (a) 25 °C, (b) 30 °C, (c) 35 °C and (d) 40 °C. Drop formation is recorded with a Uridium drop watcher high-speed camera at delays of 50 to 200 μs after firing with a jetting voltage of 110 V in a Fujifilm Dimatix Starfire SA print head.


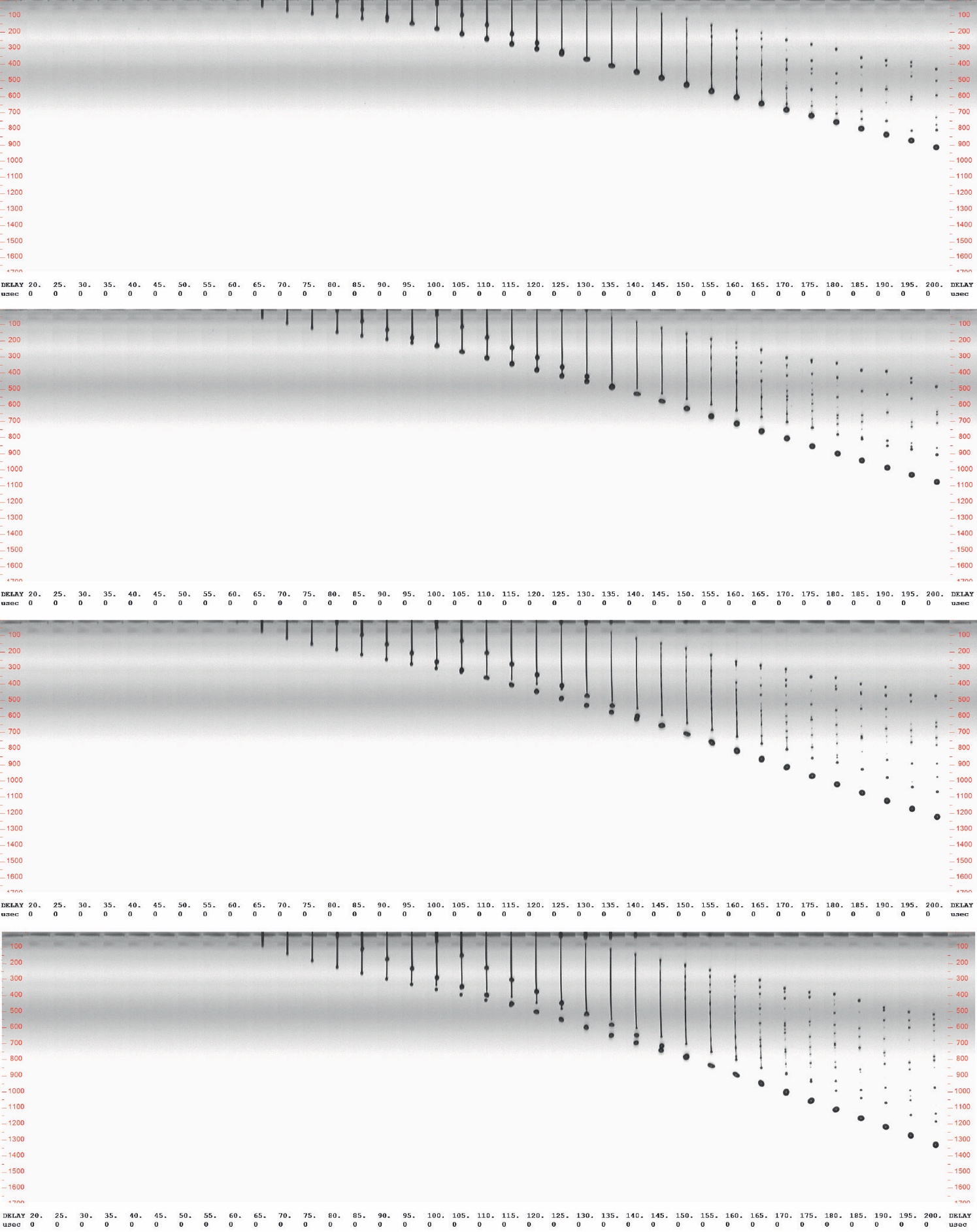

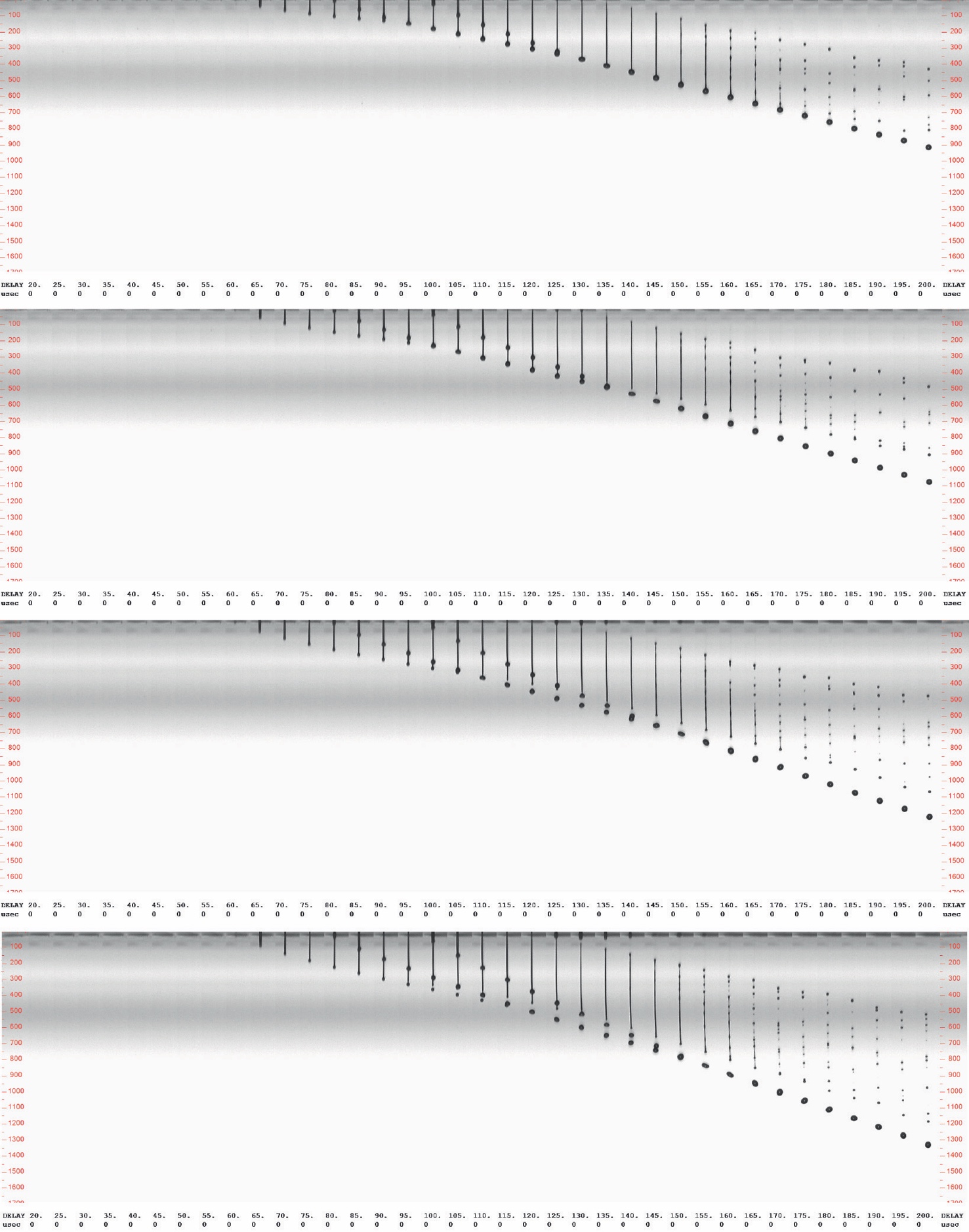


a

b

c

d

Figure S2: Representative photo sequence of drop formation of RR ink at a delay between 50 and 200 μs at varying temperatures of (a) 25 °C, (b) 30 °C, (c) 35 °C and (d) 40 °C.


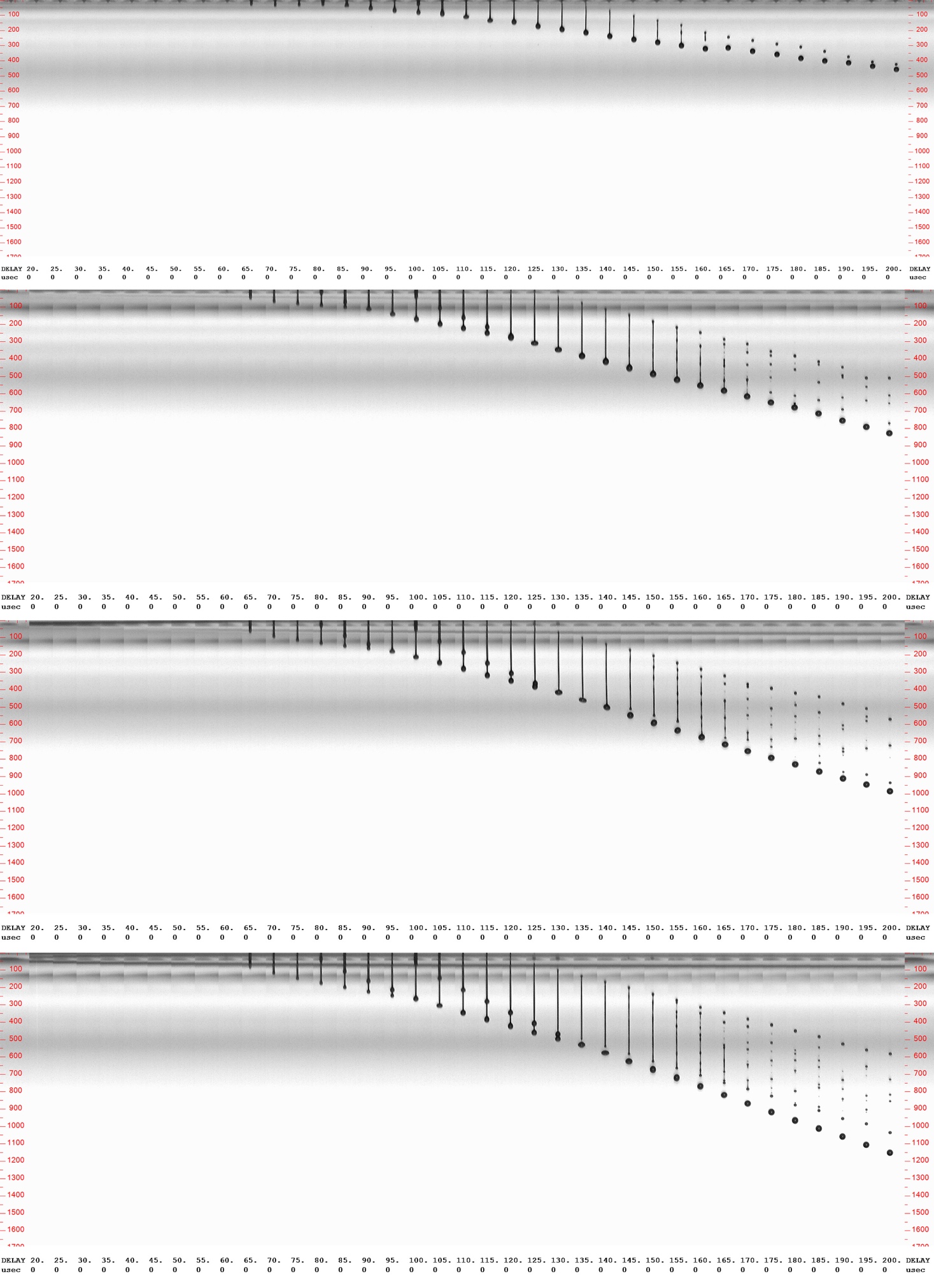

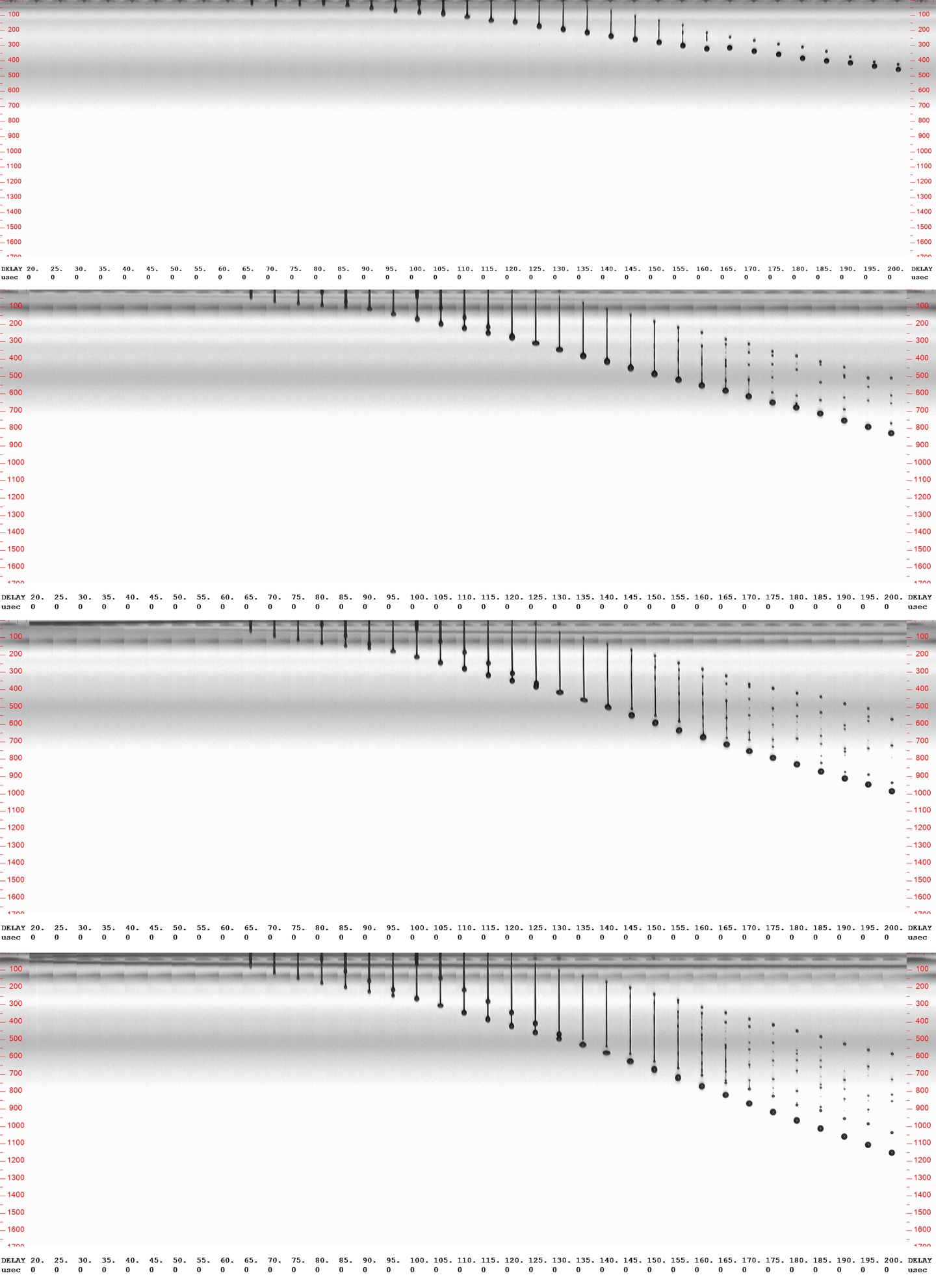


a

b

c

d

Figure S3: Representative photo sequence of drop formation of SG ink at a delay between 50 and 200 μs at varying temperatures of (a) 25 °C, (b) 30 °C, (c) 35 °C and (d) 40 °C.


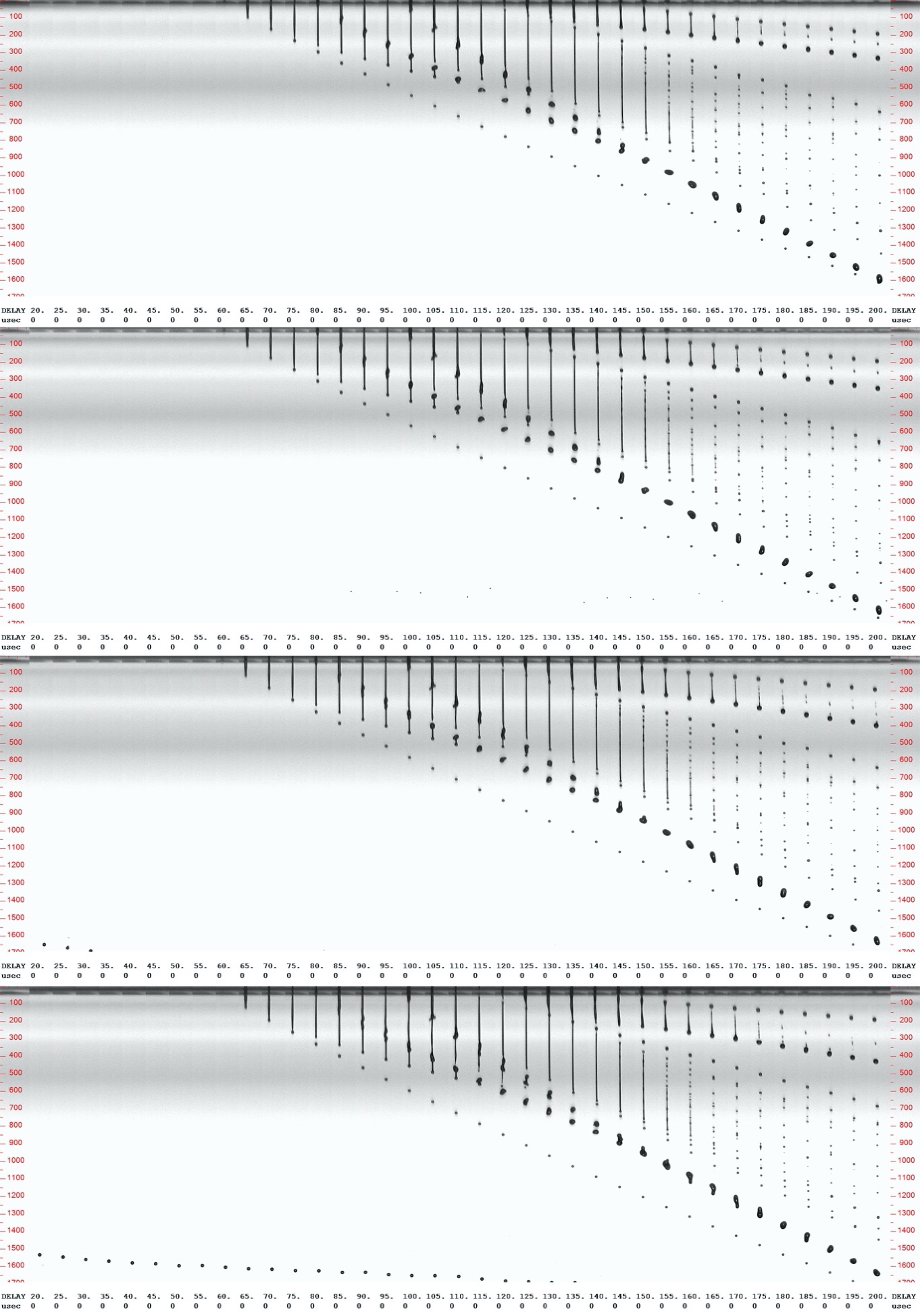

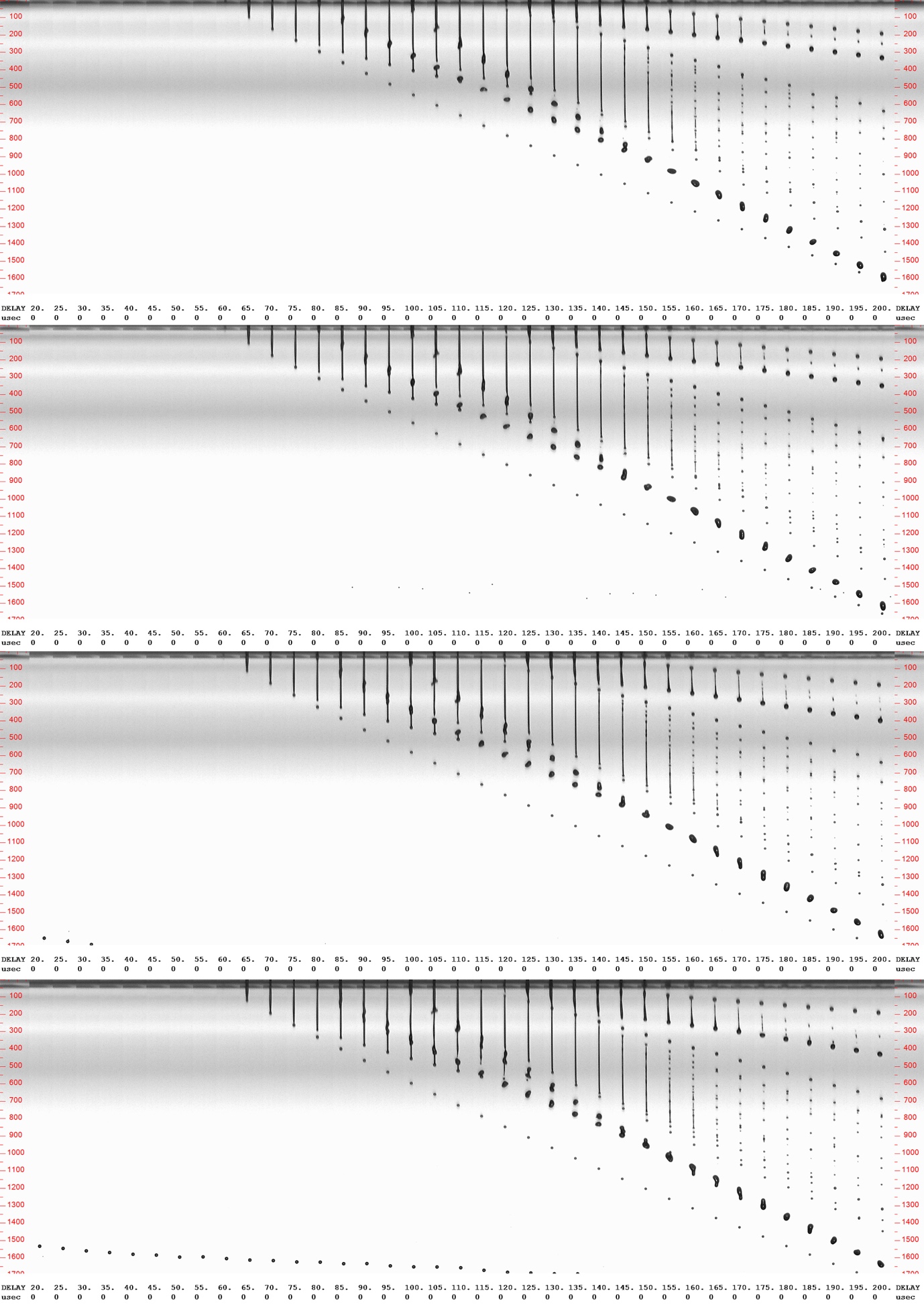


a

b

c

d

Figure S4: Representative photo sequence of drop formation of DPGMEA fluid at a delay between 50 and 200 μs at varying temperatures of (a) 25 °C, (b) 30 °C, (c) 35 °C and (d) 40 °C.

**S3. Effect of jetting voltage on drop formation of DPGMEA**

To evaluate the effect of jetting voltage on the drop formation, voltages of 70, 90, 100, 110 and 130 V have been used in jetting of the fluid. The drop formation of the standard liquid DPGMEA was recorded visually at different delays between 20 and 200 $\mu$s. Neither lower nor higher voltages than the chosen jetting voltage of 110 V could improve the jetting behavior of DPGMEA as seen in Figure 1. Satellite drops and irregular drop formation are observed for all jetting voltages.

A


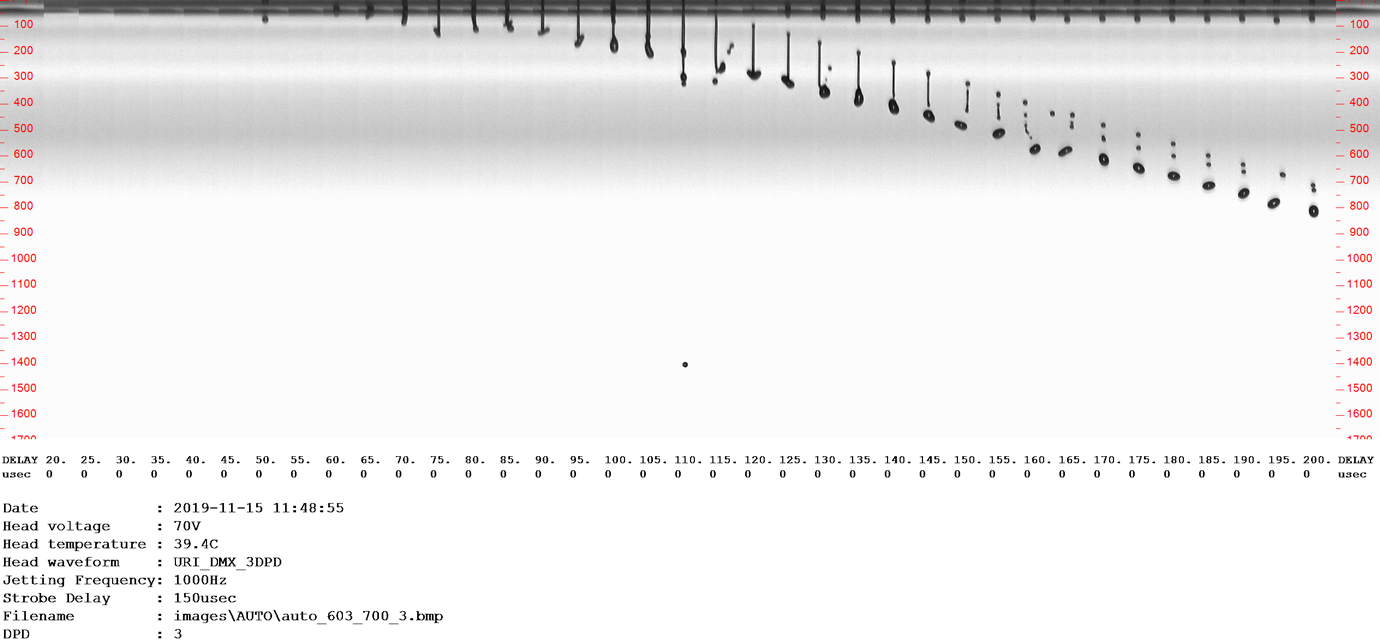


B


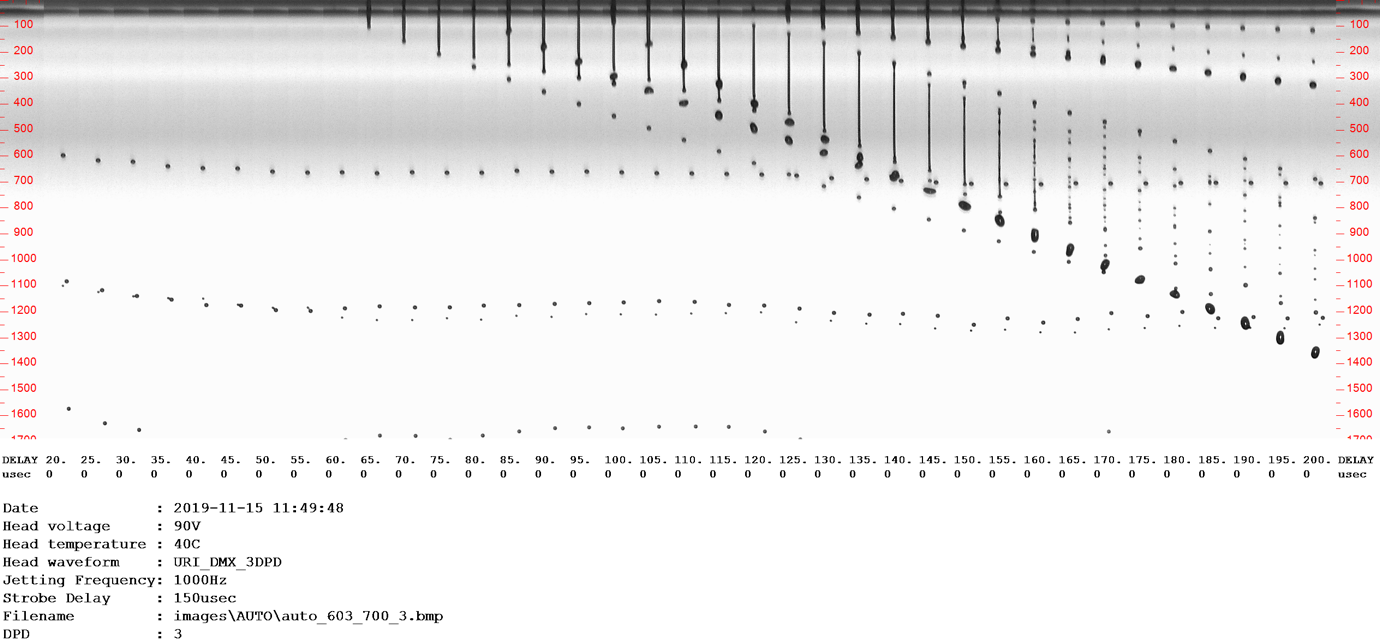


C
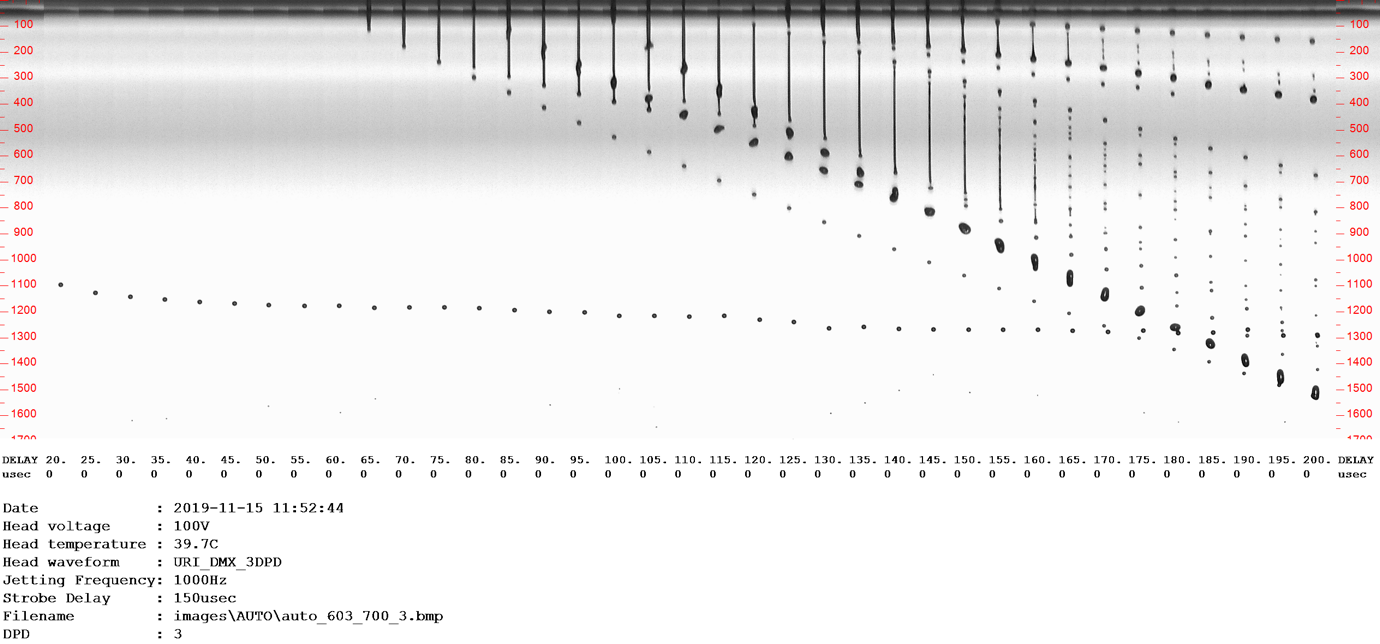


D


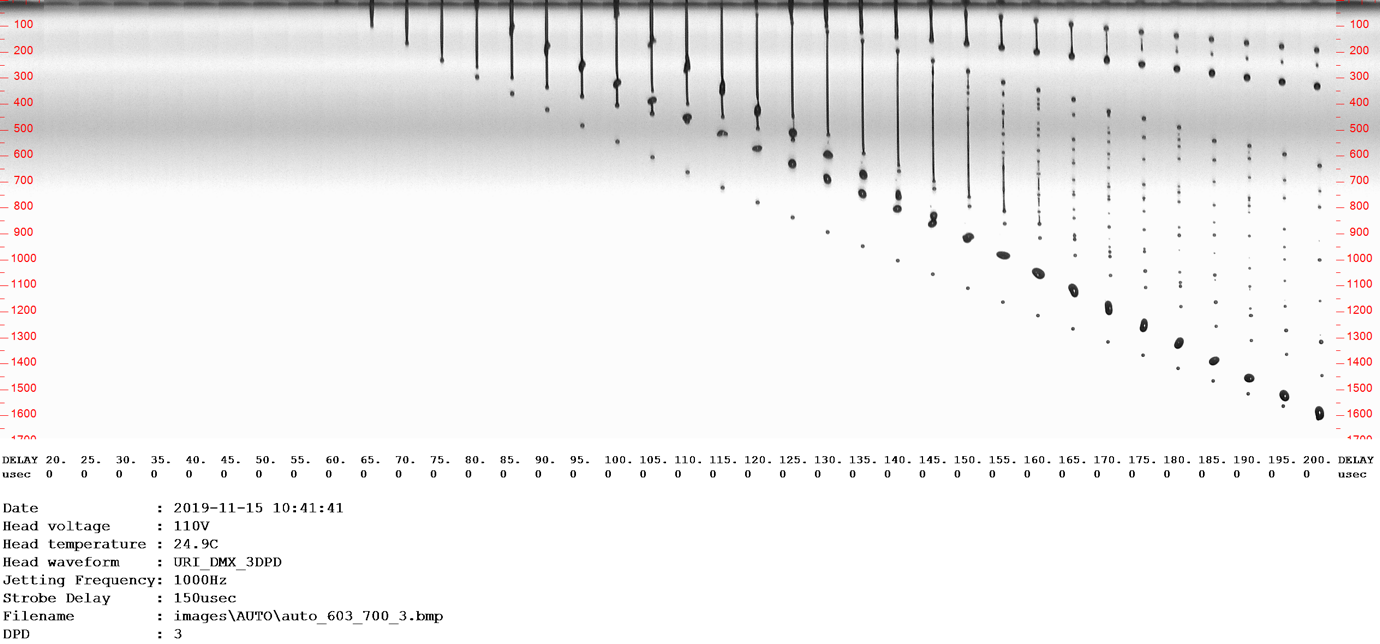


E


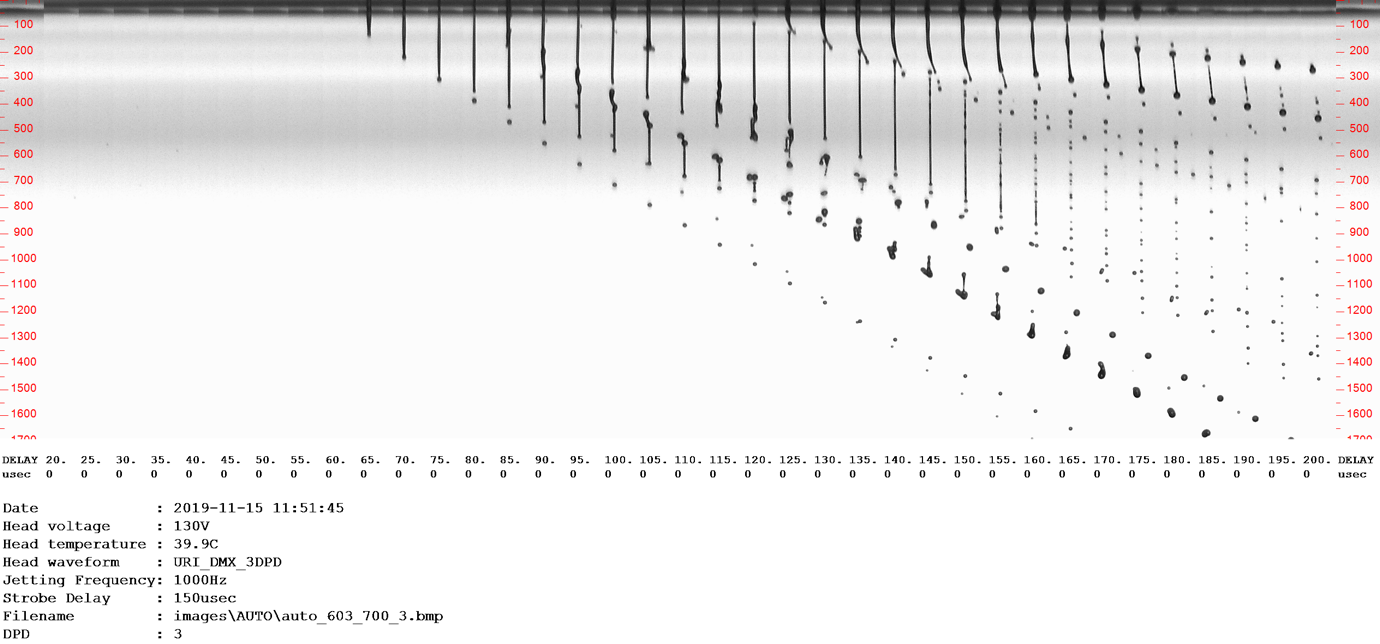


Figure S5: Representative photo sequence of drop formation of DPGMEA at a delay between 20 and 200 $\mu$s with jetting voltages of 70 V (A), 90 V (B), 100 V (C), 110 V (D) and 130 V (E).
